# A Novel Segmentation Scheme with Multi-Probability Threshold for Human Activity Recognition Using Wearable Sensors

**DOI:** 10.3390/s22197446

**Published:** 2022-09-30

**Authors:** Bangwen Zhou, Cheng Wang, Zhan Huan, Zhixin Li, Ying Chen, Ge Gao, Huahao Li, Chenhui Dong, Jiuzhen Liang

**Affiliations:** 1 School of Computer Science and Artificial Intelligence, Aliyun School of Big Data, School of Software, Changzhou University, Changzhou 213000, China; 2 School of Microelectronics and Control Engineering, Changzhou University, Changzhou 213000, China

**Keywords:** human activity recognition, threshold segmentation, slope-area method, multi-label weighted probability, machine learning

## Abstract

In recent years, much research has been conducted on time series based human activity recognition (HAR) using wearable sensors. Most existing work for HAR is based on the manual labeling. However, the complete time serial signals not only contain different types of activities, but also include many transition and atypical ones. Thus, effectively filtering out these activities has become a significant problem. In this paper, a novel machine learning based segmentation scheme with a multi-probability threshold is proposed for HAR. Threshold segmentation (TS) and slope-area (SA) approaches are employed according to the characteristics of small fluctuation of static activity signals and typical peaks and troughs of periodic-like ones. In addition, a multi-label weighted probability (MLWP) model is proposed to estimate the probability of each activity. The HAR error can be significantly decreased, as the proposed model can solve the problem that the fixed window usually contains multiple kinds of activities, while the unknown activities can be accurately rejected to reduce their impacts. Compared with other existing schemes, computer simulation reveals that the proposed model maintains high performance using the UCI and PAMAP2 datasets. The average HAR accuracies are able to reach 97.71% and 95.93%, respectively.

## 1. Introduction

With the rapid development in the fields of internet of things (IoT), human activity recognition (HAR) has gradually become a research hotspot these days. HAR provides the detection, interpretation, and recognition of different kinds of activities such as walking, running, eating, lying down, sitting, etc. Recently, numerous research works on HAR have been conducted, and most of the works are on healthcare [1,2], surveillance activities [3,4], context-aware computing [5,6], and smart home [7]. For example, in the medical industry, the accurate detection of human movement by HAR supports the development of autonomous machine-based diagnostic systems. For smart home and video surveillance, HAR applications can assist family members in remotely monitoring abnormal behaviors and the physical health conditions of the elderly and children. There mainly exist two data types, video based and sensor based, which are usually applied for HAR. Compared with the video type, the sensor type is more widely utilized since no image information of users is required, which can protect the user privacy [8].

In order to collect sensor-based data, external sensors and wearable sensors are always deployed in the HAR system [9]. For the former, the devices are fixed in a predetermined place, so the inference of activity entirely depends on the voluntary interaction between users and sensors, such as smart home environment. However, wearable sensors can support users in dealing with HAR with the data (such as accelerometer data, temperature, heart rate, etc.) collected anytime and anywhere [1]. They are widely used during HAR analysis due to their advantages of being light weight and easy to carry, having flexible installation position, and having low power consumption [5]. In recent years, the continuous development of machine learning technology has also provided efficient algorithms for HAR, such as support vector machine (SVM), K-nearest neighbor (KNN) and decision tree (DT) [10]. Here, one of the key steps is feature extraction; the extracted features include statistical features which depend on the original signals (time and frequency domain features), and cross coding (such as Fourier transform and wavelet transform) [11]. In addition, with the successful applications of deep learning technology in the field of computer vision [4], convolutional neural network (CNN), long short-term memory (LSTM), bidirectional LSTM (BLSTM), multi-layer perceptron (MLP), etc., are also introduced for sensor-based HAR [12]. It automatically extracts relevant features by constructing multi-layer deep structures [13]. Compared with the traditional classification algorithms, deep learning is able to automatically extract proper features [3]. However, a large number of samples are required for accurate analysis, and expensive hardware is indispensable to build a proper deep learning model [14].

The general process of simple activity recognition is first to identify the action segments manually from the action time series, then the HAR classifier can be generated after feature extraction and training process. However, only parts of data and labels of related actions can be known in real collected time series. There exist many challenges to identify the main human activities in a complete time series. For example, it is difficult for the trained model to classify the human activities which have not been learned before, and each segmented window may contain multiple types of activities which improve the difficulty of the classification. In addition, the starting point and the ending point of each main activity from the complete time series should be exactly found out. Additionally, body jitter and useless segments may have similar characteristics to the main activities, which decreases the accuracy of HAR. Therefore, it is an important issue to effectively identify the main activities from the time series and reject unknown activities. In [15], Gupta and Dallas (later referred to as the GD algorithm) was proposed using Relief-F and sequential forward floating search (SFFS) for feature selection. Here, naive Bayes (NB) and KNN were applied to identify six kinds of daily life activities and transition activities with a fixed window size of 6 s. In [10,16,17,18,19,20], researchers used different segmentation methods. Ref. [19] proposed to use the adaptive time window in a quasi-periodic part and fixed time window in a non-periodic part. Ref. [20] proposed an adaptive signal segmentation method to detect transition activities, and integrated it with the activity classification algorithm to overcome the limitations of the sliding window with a fixed size used in the existing work. However, these approaches require large computation, and the accuracy of the classifier still can be improved.

In this paper, a novel segmentation model based on the multi-probability threshold is proposed for complex activity recognition, and the corresponding algorithm is developed based on the characteristics of typical activities. According to the small fluctuation of static activity data, a new threshold-segmentation (TS) algorithm is proposed to find the optimal threshold according to the related measurements. Periodic-like activity has typical signal characteristics, such as peak and trough points. Through the connection of peak and trough points, the corresponding gradient area can be used to obtain the optimal threshold. Additionally, in order to identify the periodic-like activity, the slope-area (SA) filtering algorithm is applied to eliminate the abnormal points in the time series. Here, a new multi-label weighted probability model (MLWP) algorithm is proposed to obtain the probability of each activity which can be estimated by overlapping the sliding window and combining with the proposed segmentation algorithm. In addition, the threshold, $\theta_{reject}$, can support to distinguish whether the segment is the main activity or the unknown activity. The proposed method is evaluated using two common benchmarks of HAR datasets, UCI and PAMAP2. Computer simulation reveals that the proposed segmentation and recognition model significantly improves the recognition accuracy and has relatively low computational complexity. The main contributions are as follows:

- The TS algorithm is proposed according to the stationary of the static signal. A new indicator, $F_{ab}$, is estimated to identify the optimal threshold and segment from the static interval of the unknown time series.
- The SA algorithm is proposed according to the peak and trough of the periodic-like signal. Two new notions, slope and area, are employed to eliminate the abnormal points which support to identify the suspected periodic-like interval of the unknown time series.
- Combined with the pre-segmentation results, a multi-probability threshold recognition model is proposed, which not only substantially improves the accuracy of HAR, but also effectively distinguishes the useless segments in the complex continuous time series.

The remainder of this paper is organized as follows. Section 2 provides the related work of HAR. Section 3 describes the proposed multi-probability threshold recognition model and the segmentation algorithms. Section 4 introduces the HAR data set and shows the performance evaluation of the proposed scheme. Finally, Section 5 summarizes the paper and lists the future work.

## 2. Related Work

### 2.1. Human Activity Recognition

Recent HAR researches focus on typical activities (e.g., walking, standing, sitting, and running). However, human daily activities are always complex and continuous, which may include transition and atypical actions. Esfahani et al. showed that location-aware multi-sensors (PAMS) can significantly improve the classification accuracy of HAR [7]. Gyllensten et al. [21] used traditional machine learning technologies to classify static and dynamic actions in human daily life. Wan et al. [11] applied deep learning methods to identify human activities, including CNN, LSTM and other methods. Gyroscopes can also be used for HAR. It has been proved that the use of gyroscopes and accelerometers can improve the recognition performance [22]. In [23], the hidden Markov model (HMM) was introduced to detect feeding activities with the collected data of acceleration and angular velocity of the arms, and the accuracy rate reached 84.3%.

In [24], the researchers proposed a lightweight CNN using Lego filters for HAR, which can greatly reduce the cost of memory and computation compared with traditional CNN. Ref. [25] introduced the mixed channel and time attention mechanism into CNN, which enhanced the interpretability. CondConv [26] was employed to replace the standard convolution procedure in CNN. The performance of the model can be improved by increasing the number of experts. Yang et al. [27] quantified the weight and adopted dynamic fusion strategy for different types of activities, which achieved good results on multiple data sets and greatly saved memory. Since deep learning methods require a large number of samples and expensive hardware to train the model, the shallow learning method is mainly focused on in this paper. Experiments show that it can also achieve good classification results with fewer computing resources.

### 2.2. Signal Segmentation

HAR can be essentially simplified as a multivariate time series classification problem [4]. The signal is divided into different fragments by using segmentation methods, and then these fragments are mapped to specific activities [3]. In [18], the researchers proposed a method to dynamically adjust the window size based on entropy for activity recognition, but it did not consider the transition actions. Ref. [28] applied a data stream segmentation algorithm to adjust the window size according to whether the data value is stable. These algorithms are very sensitive to noise. Therefore, it is necessary to preprocess signals before recognition. There exist many kinds of filters, such as the Butterworth filter [28], Chebyshev filter, Bessel filter and Elliptic filter [29]. In [9], researchers used the Butterworth filter to process acceleration data and achieved good results. Referring to the extraction algorithm of fundamental tone in speech signal processing, an adaptive time window method was employed to accurately extract features from periodic-like signals for HAR [19]. Experiment showed that it has a good recognition rate for dynamic and static activities. A symbol-based segmentation method [30] was proposed to detect the gait phase and transmit important dynamic information from the accelerometer signal. Here, a symbol-based symmetry index was introduced to replace the traditional one.

**Figure 1:**
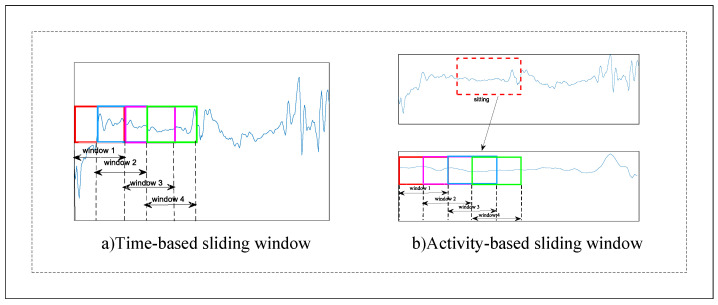
Two different segmentation methods of sliding window. (**a**) Time-based, and (**b**) Activity-based.

As shown in Figure 1, sliding window is a typical segmentation method to solve the HAR problem [5], which can be mainly divided into two types: time based and activity based. The time-based type is the window segmentation of the original signal. Jorge-L et al. [17] proposed the transition-aware human activity recognition (TAHAR) system architecture, which has greatly improved the recognition of transition actions in UCI [9], PAMAP2 [31] and REALDISP dataset. Noor et al. [20] used the adaptive window segmentation method to solve the limitation of the fixed window segmentation in UCI [9] dataset. Here, the window was adaptively expanded according to the probability of the action in the window. Activity-based segmentation is the window segmentation of the data segments of each activity. Fidad et al. studied the recognition effect of different lengths of windows on short-term activities (sitting, standing and transition) and long-term activities (walking, upstairs and downstairs) and used a self-collected dataset, for which subjects wore a tri-axial accelerometer on their waist [10]. Since the gait recognition performance decreases with the change of walking speed, Sun et al. [16] proposed a gait segmentation method based on adaptive speed, and the threshold was generated by single match. The ZJU-GaitAcc public dataset and self-collected dataset were utilized in the comparative experiment. For these two sliding window segmentation methods, the activity-based type does not need to consider the situation of useless segments or multiple activities in one window, which can achieve better accuracy. However, for the complex and continuous time series of HAR, data contain useless segments, and the starting point of the activity is unknown. This paper adopts the time-based sliding window segmentation method.

## 3. The Proposed Scheme

### 3.1. Problem Formalization

In this paper, assume that volunteers have *k* sensors in different parts of their bodies, while all sensors have the same sampling frequency and emission time synchronization. Usually, wearable sensors, such as smart phones and the inertial measurement unit (IMU), are equipped with accelerometers, gyroscopes and magnetometers. Each sensor can generate multi-dimensional signals (for example, accelerometers can generate three-dimensional signals along the *x*-axis, *y*-axis and *z*-axis), and the signals generated by all sensors can be expressed as a multi-dimensional time series *T* as shown in Equation (1). Here, $T_{t}$ represents the 1×k output vector by the *k* sensors at time *t*, and $T_{kt}$ represents the output data of the *k*th sensor at time *t*, so *T* is a matrix with size t×k.

(1) $$T={\left[T_{1},T_{2},\ldots ,T_{t}\right]}^{T}\;with\;T_{t}=\left[T_{1t},T_{2t}\ldots ,T_{kt}\right]$$

**Figure 2:**
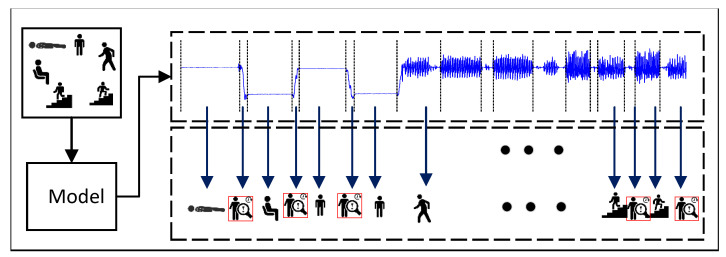
Problem formalization of HAR.

As shown in Figure 2, it is assumed that volunteers perform a total of *N* different daily activities during time *t*, including some useless segments caused by body jitter or manually unmarked segments. Let $A=\left\{\left.g_{1},g_{2},\ldots ,g_{N},g_{\tau }\right\}\right.$ represent the whole recognition set of activities, where $g_{\tau }$ is the category of useless segments. Then the complex HAR problem can be described as follows: given an unknown *S*, find the various activities occurring in *S* and identify their corresponding starting and ending positions. The mathematical description is shown in Equation (2), where $S_{u_{i}r_{i}}$ represents the sequence segment of the ith activity segment from time $u_{i}$ to $r_{i}$, and *o* is the number of activity segments in the time series.

(2) $$\begin{array}{cc} \; & \;\overset{o}{\underset{i=1}{⋃}}S_{u_{i}r_{i}}\;=\;S\\ \; & s.t.\;S_{u_{i}r_{i}}\in A\;=\;\left\{\left.g_{1},g_{2},\ldots ,g_{N},g_{\tau }\right\}\right.\\ \; & \;1\leq u_{i}\leq r_{i}\leq t\;and\;1\leq i\leq o \end{array}$$

### 3.2. The Proposed Framework

**Figure 3:**
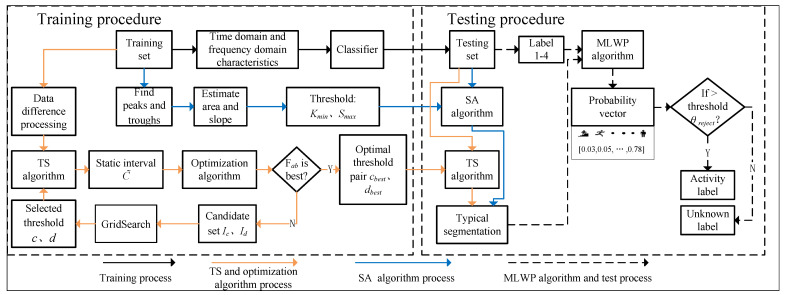
Framework diagram of the proposed scheme.

Figure 3 shows an overall framework which is proposed to segment and identify unknown time series with multiple activities. The black, orange, blue, and the black dotted arrow represent the training procedure, TS algorithm and its optimization, SA algorithm, and MLWP algorithm and testing procedure, respectively. Therefore, the framework mainly includes four procedures:

- The training set is segmented by sliding window based on activity, and the corresponding time–frequency domain features are extracted manually. The recognition model is trained by traditional classifiers (SVM, DT, NB, etc.).
- For the training set, TS and its optimization algorithms are used to find the optimal threshold parameters, *c_best_* and *d_best_*, and apply them to the testing set to identify the suspected static segmentations in the time series.
- For the training set, the peak–trough method is applied to estimate the related slope, *K_min_*, and area, *S_max_*. The SA algorithm is used to detect and eliminate the outliers, and the suspected periodic-like segmentations in the testing set can be determined.
- The testing set is segmented according to the method of overlapping sliding window and feature extraction, and multi-class labels are generated by training the model. Combined with the basic activity segmentations identified before, the probability vector of each window can be obtained by the MLWP algorithm. Correct activity category and unknown ones of the window can be distinguished by $\theta_{reject}$.

Section 3.3 shows the data preprocessing. In Section 3.4, the TS algorithm and optimization algorithm are explained in detail, and the optimal threshold *c_best_* and *d_best_* are obtained. Section 3.5 shows how to segment periodic-like interval and detailed description of the exclusion of outliers. After the test sample is pre-segmented, activity recognition was carried out according to MLWP  algorithm in Section 3.6.

### 3.3. Filtering and Feature Extraction

In the real environment, the signal generated by the sensor usually contains noise, and even the data can be lost. Therefore, it is necessary to preprocess the raw signal first. In order to reduce the interference of random noise on the signal, the median filter and third-order Butterworth filter are employed to handle the original signal. Here, the acceleration and angular velocity data are utilized to extract features in order to improve the HAR performance [16]. Six new sets of data, $A_{x}^{{}^{\prime }}$, $A_{y}^{{}^{\prime }}$, $A_{z}^{{}^{\prime }}$, $G_{x}^{{}^{\prime }}$, $G_{y}^{{}^{\prime }}$ and $G_{z}^{{}^{\prime }}$, are generated by obtaining derivatives with respect to the original data (including $A_{x}$, $A_{y}$, $A_{z}$, $G_{x}$, $G_{y}$ and $G_{z}$) from each sensor. In addition, the Euclidean norm of the original acceleration, $R_{A}$, and angular velocity data, $R_{G}$, can be calculated to obtain two new sets of data. Therefore, in total, 14×k sets of data are obtained, which include 6 sets of original data and 8 sets of generated new data, where *k* is the number of sensors. The sliding window method is used to extract 7 time domain features (mean value, standard deviation, mode, maximum, minimum, skewness and kurtosis) and three frequency domain features (gravity frequency (the weighted average of the amplitude of the power spectrum), frequency variance and mean square frequency) from each set of data of each window so that each sliding window can obtain a total of 140×k sets of statistical features. The initial feature set and descriptions of 14 signals of HAR are listed in Table 1 and Table 2.

### 3.4. Static Segmentation

Human activity can be divided into static, dynamic and transition actions. Compared with the dynamic and transition action, static action has a little rate of change. Therefore, the difference of the signals can be clearly reflected through acceleration and angular velocity.

**Figure 4:**
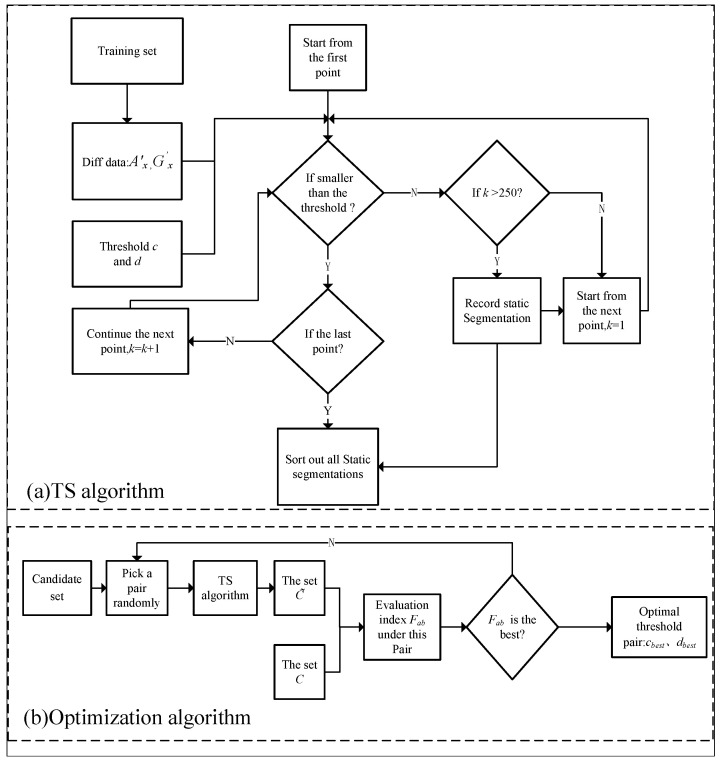
Flow chart of the proposed TS algorithm. (**a**) TS algorithm, and (**b**) Optimization algorithm.

The signals of acceleration and angular velocity are differential processed, respectively, and the static segmentations in the whole time series can be identified by setting the threshold. As shown in Figure 4a, a set of thresholds is randomly selected from the candidate value pairs to obtain the corresponding static segmentation $\tilde{C}$ by the proposed TS algorithm. Figure 4b illustrates the selection procedure of the threshold pair using the grid search approach. The evaluation indicator *F_ab_* can be estimated through comparing $\tilde{C}$ from Figure 4a with *C* labeled manually. The best threshold pair, *c_best_* and *d_best_*, are finally obtained if *F_ab_* is optimized. A detailed estimation of *F_ab_* is provided below.

For a complex time series, the starting point of the focused activity is often manually identified [7]. $C=\left\{S_{u_{1}r_{1}},S_{u_{2}r_{2}},\ldots ,S_{u_{i}r_{i}},\ldots ,S_{u_{K}r_{K}}\right\}$, where $\;1\leq u_{i}<r_{i}\leq t\;\mathrm{and}\;1\leq i\leq K$. *C* is the set of static segmentations manually identified. $S_{u_{k}r_{k}}$ represents the static segmentation from time *uk* to *rk*, while *K* represents the number of static segmentations manually identified. After differential processing of the time series data, it can be found that the difference between the static segmentations is relatively small during [0, *g*], where *g* is the gravity acceleration. Referring to the grid search method, this paper exhaustively traverses all the hyper-parameter combinations in order to select the optimal set as the final results. The purpose of TS algorithm is to find out the optimal threshold *c_best_* and *d_best_* to cut out the optimal static segmentations. It is assumed that the thresholds *c* and *d* both have *z* groups of candidate parameters, and listed as 1×z one-dimensional matrices, *I_c_* and *I_d_*, respectively. *I_c_* and *I_d_* can generate z×z candidate values. According to Figure 4a, the static segmentations with different candidate values can be identified, and the related optimal threshold can be finally obtained according to Figure 4b. Let $\tilde{C}=\left\{\left.S_{\tilde{u_{1}}\tilde{r_{1}}},S_{{\tilde{u}}_{2}{\tilde{r}}_{2}},\ldots ,S_{{\tilde{u}}_{i}{\tilde{r}}_{i}},\ldots ,S_{{\tilde{u}}_{\tilde{K}}{\tilde{r}}_{\tilde{K}}}\right\}\right.$, where $\;1\leq \tilde{u_{i}}<\tilde{r_{i}}\leq t\;\mathrm{and}\;1\leq i\leq \tilde{K}$ be a set of static segmentations identified using candidate thresholds *c* and *d*. Here, $\tilde{K}$ represents the number of static segmentations identified by the TS algorithm, and  $S_{{\tilde{u}}_{i}\tilde{r_{i}}}$ denotes a static segmentation from time ${\tilde{u}}_{i}$ to time $\tilde{r_{i}}$.

**Figure 5:**
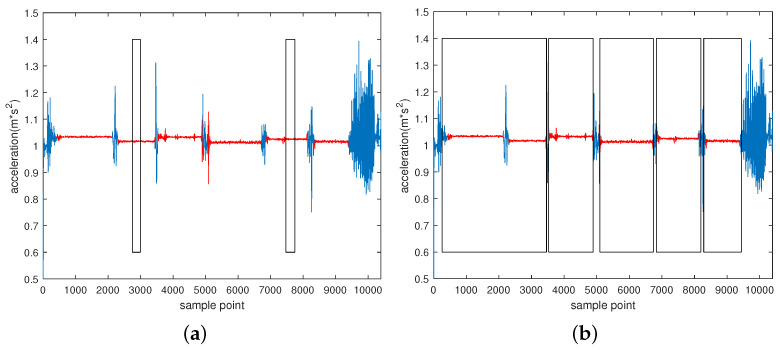
Segmentation results under different thresholds. (**a**) small threshold, and (**b**) big threshold.

In order to find the optimal thresholds in the training samples, the algorithm should clearly determine the optimized static segmentations, while the TS algorithm does not mix up with the segmentations of other types of activities. As obtained in Equations (3) and (4), $S_{a}$ denotes the total number of sampling points in static segmentations labeled manually, while $S_{b}$ is the total number of sampling points in static segmentations identified by the TS algorithm with the candidate pairs of thresholds. Here, $u_{i}$ and $r_{i}$ represent the starting and ending points of the *i*th static segmentation in set *C*, respectively. Similarly, $\tilde{u_{j}}$ and $\tilde{r_{j}}$ are the starting and ending points of the *j*th static segmentation in set $\tilde{C}$, respectively. $S_{ab}$ represents the number of sampling points in the overlapping areas of the static segmentations identified by the TS algorithm and the labels. $S_{ab}/S_{a}$ represents the proportion of all static intervals that are correctly split. $S_{ab}/S_{b}$ represents the proportion of correct segmentation in the interval segmented by the TS algorithm. In order to divide the interval to be both right and complete, $S_{ab}/S_{a}$ and $S_{ab}/S_{b}$ should be as big as possible. As shown in Figure 5, the red parts are the manually labeled static segmentations, and the black rectangular boxes are the static segmentations identified by the TS algorithm. In Figure 5a, most static intervals are not split using a very small threshold, then $S_{a}/S_{ab}$ is small. In Figure 5b, the transition actions are contained in $S_{b}$ which result in $S_{b}/S_{ab}$ being smaller. Therefore, there exists a trade-off between these two requirements. The F1-score is an indicator used to measure the accuracy of binary classification model in statistics, which considers the accuracy and recall of the classification model at the same time. According to the logic of F1-score, $F_{ab}$ is calculated in Equation (5).

**Figure 6:**
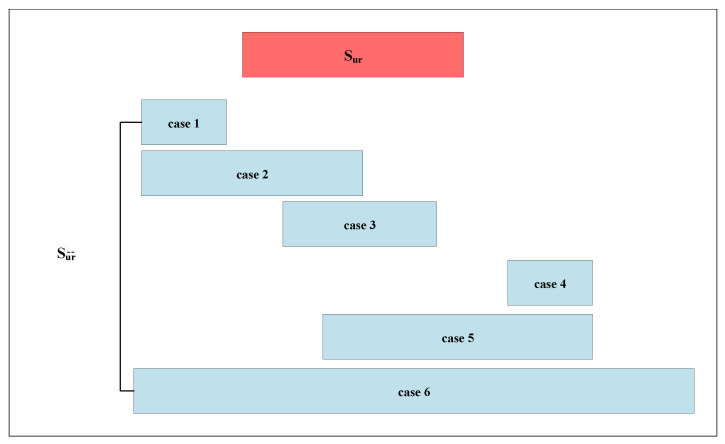
The relative position of the interval cut by the algorithm and the manual annotation interval.

As shown in Figure 6, the red and blue parts represent the static segmentations identified manually and by the TS algorithm, respectively. It can be found that there exist only four cases in which overlapping occurs among the total six cases of the relative positions between two kinds of static segmentations.

For cases 1–3, it can be seen that the ending point of the segmentations using the TS algorithm is smaller than those manually labeled. If the ending point of the blue parts is smaller than the starting point of the red part, there is no overlapping area. Therefore, $max\left(0,\left(\tilde{r_{j}}-u_{i}\right)\right)/|\tilde{r_{j}}-u_{i}|$ is used to eliminate case 1. Additionally, Figure 6 shows that the overlapping area can be obtained as follows: $\min\left(\tilde{r_{j}},r_{i}\right)-\max\left(u_{i},\tilde{u_{j}}\right)+1$. For cases 4–6, similarly, the ending points of the blue parts are greater than the red part. Then, the total overlap point number of *C* and $\tilde{C}$ is obtained as $max\left(0,\left(\tilde{r_{j}}-u_{i}\right)\right)/|\tilde{r_{j}}-u_{i}|\times \left(\min\left(\tilde{r_{j}},r_{i}\right)-\max\left(u_{i},\tilde{u_{j}}\right)+1\right)$. In summary, the overall calculation can be estimated in Equation (6).

(3) $$S_{a}=\sum_{i=1}^{K}\left(r_{i}-u_{i}+1\right)$$

(4) $$S_{b}=\sum_{j=1}^{K}\left(\tilde{r_{j}}-\tilde{u_{j}}+1\right)$$

(5) $$F_{ab}=2\times {\frac{s_{a}\times s_{b}}{(s_{a}+s_{b})\times s}}_{ab}$$

(6) $$S_{ab}=\left\{\begin{array}{c} \sum_{i=1}^{K}\sum_{j=1}^{\tilde{K}}[\frac{\max(0,\left(\tilde{r_{j}}-u_{i}\right))}{|\tilde{r_{j}}-u_{i}|}\times (\min\left(\tilde{r_{j}},r_{i}\right)-\max\left(u_{i},\tilde{u_{j}}\right)\\ +1)],\tilde{r_{i}}\leq r_{j};\\ \sum_{i=1}^{K}\sum_{j=1}^{\tilde{K}}[\frac{\max(0,\left(r_{i}-\tilde{u_{j}}\right))}{|r_{i}-\tilde{u_{j}}|}\times (\min\left(\tilde{r_{j}},r_{i}\right)-\max\left(u_{i},\tilde{u_{j}}\right)\\ +1)],{\tilde{r}}_{i}>r_{j}. \end{array}\right.$$

Algorithm 1 gives the detailed procedures of the proposed TS algorithm.
**Algorithm 1** The proposed TS algorithm.**Input:***C, $I_{c}$, $I_{d}$*, $A_{x}^{{}^{\prime }}$, $G_{x}^{{}^{\prime }}$.**Output:***$c_{best}$, $d_{best}$***initialization:***$c_{best}$, $d_{best}$, $f_{best}$* = 01:**function**Segmentation($A_{x}^{{}^{\prime }}$, $G_{x}^{{}^{\prime }}$, *c*, *d*)2:    *i* = 1, *k* = 03:    **while** *i* <= length($A_{x}^{{}^{\prime }}$) **do**4:        **if** $A_{x}^{{}^{\prime }}\left(i\right)<c$ && $G_{x}^{{}^{\prime }}\left(i\right)<d$ **then**5:           *k* = *k* + 1, *i* = *i* + 16:        **else**7:           **if** ($A_{x}^{{}^{\prime }}\left(i\right)$>c ∥ $G_{x}^{{}^{\prime }}\left(i\right)$>d) && k>250 **then**8:               $\left[i-k,i+1\right]=\tilde{C}$, *k* = 0, *i* = *i* + 19:           **end if**10:        **end if**11:    **end while**12:    **return** $\tilde{C}$13:**end function**14:**function**compare(x,y)15:    Calculate $S_{a}\left(x\right)$ using Equation (3).16:    Calculate $S_{b}\left(y\right)$ using Equation (4).17:    Calculate $S_{ab}\left(x,y\right)$ using Equation (6).18:    Calculate $F_{ab}$ using Equation (5).19:    **return** $F_{ab}$20:**end function**21:**for***i* from 1 to *z* **do**22:    **for** *j* from 1 to *z* **do**23:        $\tilde{C}=Segmentation\left(A_{x}^{{}^{\prime }},G_{x}^{{}^{\prime }},I_{c}\left(i\right),I_{c}\left(j\right)\right)$24:        $F_{ab}=compare\left(C,\tilde{C}\right)$25:        **if** $F_{ab}>f_{best}$ **then**26:           $c_{best}=I_{c}\left(i\right),d_{best}=I_{d}\left(i\right)$27:        **end if**28:    **end for**29:**end for**30:**return**$c_{best},d_{best}$

### 3.5. Periodic-like Interval Segmentation

**Figure 7:**
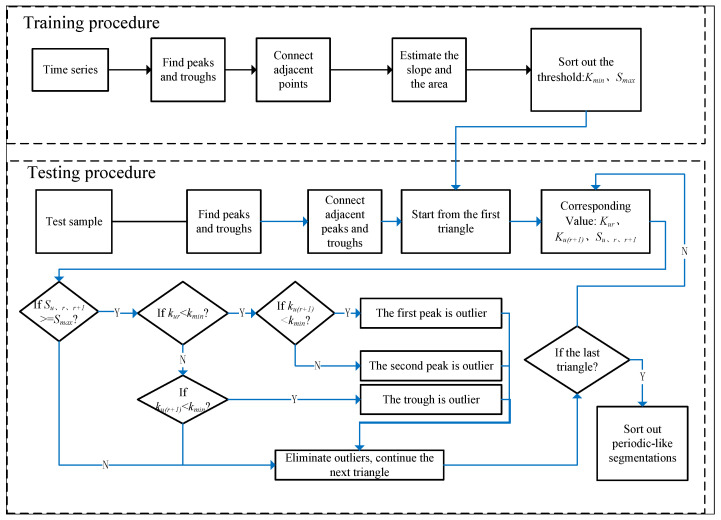
The flow chart of the proposed SA algorithm.

Periodic-like activity usually takes a long time. Here, peaks and troughs can clearly reflect the characteristics of periodic signals. Generally, the horizontal spacing distance between the peaks and troughs are half of the human activity cycle. For a complex time series, the periodic-like action segmentations can be identified by finding peaks and troughs. However, the transition activity between two static activities and the jitter of human body has the probability to generate abnormal peaks and troughs, which may bring serious impacts to HAR. Therefore, the SA algorithm is applied to eliminate these abnormal points. The area of the line connecting two adjacent peaks and the troughs between them will be much larger than the normal area, so the abnormal points can be preliminarily found according to the calculated area. However, the abnormal points cannot be accurately identified only by using the area. Another notion, slope, is introduced. Since for the abnormal points, the slope of the line connecting two adjacent peaks and the troughs between them is much smaller than the normal slope, the abnormal points can be further filtered by the slope. The flowchart of the proposed SA algorithm is shown in Figure 7. The training part lists the peaks and troughs and connects the adjacent peaks and troughs to estimate the threshold slope and area, then finds the minimum slope value and maximum area value stored as $k_{min}$ and $S_{max}$. The test data set repeats the procedure to calculate the related slope and area which are used to compare with $k_{min}$ and $S_{max}$. After eliminating the abnormal points, the periodic-like segmentations can be cut out from the time series.

**Figure 8:**
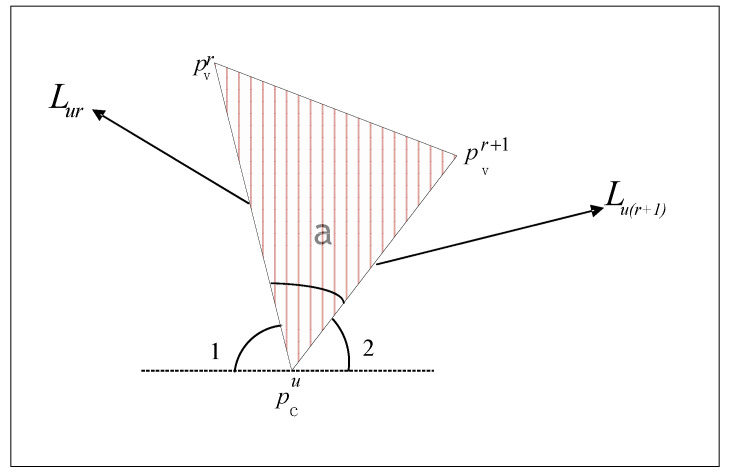
Triangle diagram of the lines connecting adjacent peaks and the related trough.

Let $P_{v}=\left\{p_{v}^{1},p_{v}^{2},p_{v}^{3},\ldots ,p_{v}^{m}\right\}$ and $P_{c}=\left\{P_{c}^{1},P_{c}^{2},P_{c}^{3},\ldots ,P_{c}^{n}\right\}$ be the set of peak and trough points in the periodic-like segmentations in the training sample, respectively. Here, m and n are the number of peak and trough points, respectively. Suppose $k_{ur}$ and $L_{ur}$ are the absolute value of the slope and the length of the line connecting between the peak point ($p_{v}^{r}$) and the trough point ($p_{c}^{u}$), respectively. Similarly, $k_{u(r+1)}$ and $L_{u(r+1)}$ are the absolute value of the slope and length of the line connecting between the peak point ($p_{v}^{r+1}$) and the trough point ($p_{c}^{u}$). Here, $P_{v}^{r}<P_{c}^{u}<P_{v}^{r+1},1\leq u\leq n,1\leq r<m$. It is necessary to calculate the area of the lines connecting three points, $p_{c}^{u}$, $p_{v}^{r+1}$, and $p_{v}^{r}$, where the triangle area is $S_{u,r,r+1}=\frac{1}{2}L_{ur}\times L_{u(r+1)}\times \sin a$, and a is the angle of the lines connecting three points, as shown in Figure 8. The slope of point $p_{c}^{u}$ and $p_{v}^{r}$, $k_{ur}$ can be obtained as shown in Equation (7). Here, $x_{p_{v}^{r}}$ and $y_{p_{v}^{r}}$ represent the corresponding number of sampling points and the acceleration value of point $p_{v}^{r}$, respectively. Let $1/k_{ur}$ and $1/k_{u(r+1)}$ be the tangent values of ∠1 and ∠2. The tangent values of *∠*a can be obtained, as shown in Equation (8). According to tan *a* obtained above, the corresponding sin *a* can be obtained using Equation (9). Then the triangle area, $S_{u,r,r+1}$ can be calculated by Equation (10).

(7) $$k_{ur}=\frac{y_{p_{v}^{r}}-y_{p_{c}^{u}}}{x_{p_{v}^{r}}-x_{p_{c}^{u}}}$$

(8) $$\tan a=\tan\left(\pi -\left(1+2\right)\right)=-\tan\left(1+2\right)=\frac{1/k_{ur}+1/k_{u(r+1)}}{1/(k_{ur}\times k_{u(r+1)})-1}=\frac{k_{ur}+k_{u(r+1)}}{1-k_{ur}\times k_{u(r+1)}}$$

(9) $$\sin a=\sqrt{\frac{\tan^{2}a}{\tan^{2}a+1}}=\sqrt{\frac{{\left(k_{ur}+k_{u(r+1)}\right)}^{2}}{{\left(k_{ur}+k_{u(r+1)}\right)}^{2}+{\left(k_{ur}\times k_{u(r+1)}-1\right)}^{2}}}$$

(10) $$S_{u,r,r+1}=\frac{L_{u(r+1)}\times L_{ur}}{2}\times \sqrt{\frac{{\left(k_{ur}+k_{u(r+1)}\right)}^{2}}{{\left(k_{ur}+k_{u(r+1)}\right)}^{2}+{\left(k_{ur}\times k_{u(r+1)}-1\right)}^{2}}}$$

Then the normal values of slope and area are estimated to determine the threshold. Finally, the abnormal points in complex time series are eliminated by the proposed SA algorithm, and the periodic-like segmentations can be clearly identified. Let $\tilde{P_{v}}=\left\{\tilde{p_{v}^{1}},\tilde{p_{v}^{2}},\tilde{p_{v}^{3}},\ldots ,\tilde{p_{v}^{\tilde{m}}}\right\}$ and $\tilde{P_{c}}=\left\{\tilde{P_{c}^{1}},\tilde{P_{c}^{2}},\tilde{P_{c}^{3}},\ldots ,\tilde{P_{c}^{\tilde{n}}}\right\}$ be the sets of the peak and trough points in the test sample, respectively. The corresponding $k_{\min}$ and $s_{\max}$ are calculated according to Equations (7) and (10).

Algorithm 2 lists the detailed steps of the proposed SA algorithm. Output, *D*, is the set of the peak and trough points without abnormal points.
**Algorithm 2** The proposed SA algorithm.**Input:**$K_{min}$, $S_{max}$, $P_{c}$, $P_{v}$, $\tilde{P_{c}}$, $\tilde{P_{v}}$**Output:***D*1:**function**GetSlopeArea($P_{c}$, $P_{v}$)2:    Calculate $K(P_{c},P_{v})$ using Equation (7).3:    Calculate $S(P_{c},P_{v})$ using Equation (8)–(10).4:    **return** K,S5:**end function**6:**function**eliminate($K,S,K_{min},S_{max}$)7:    *i* = 18:    **while** *i* > length(s) **do**9:        **if** $S>S_{max}$ **then**10:           **if** $K\left(i\right)<K_{min}$ **then**11:               **if** $K\left(i+1\right)<K_{min}$ **then**12:                   $outlier=P_{i},i=i+1$13:               **else**14:                   $outlier=T_{i+1},i=i+1$15:               **end if**16:           **else**17:               **if** $K\left(i+1\right)<K_{min}$ **then**18:                   $outlier=T_{i},i=i+1$19:               **end if**20:           **end if**21:        **end if**22:    **end while**23:    **return** outlier24:**end function**25:$K,S=GetSlopeArea(\tilde{P_{c}},\tilde{P_{v}})$26:$outlier=eliminate(K,S,k_{min},s_{max})$27:${\tilde{P_{c}}}^{{}^{\prime }}=\tilde{P_{c}}-outlier$28:${\tilde{P_{v}}}^{{}^{\prime }}=\tilde{P_{v}}-outlier$29:$D={\tilde{P_{c}}}^{{}^{\prime }}+{\tilde{P_{v}}}^{{}^{\prime }}$30:**return***D*

### 3.6. Multi-Label Weighted Probability Model (MLWP)

For complex HAR using the sliding window method, the window is prone to make classification errors at the boundaries of different actions or at the segmentations due to body jitter. Even worse, some useless segmentations may be classified into major activities. If a sliding window of a small size is used to reduce the number of sampling points at the boundary while improving the recognition rate, this may lead to the loss of the basic characteristics of other activities. Therefore, window overlap is a better solution. Let the sliding window be overlapped by q%, which means each sub-window produces ⌈1/(q%)⌉ labels. When the overlapping sliding window method is used for classification and recognition, the sub-windows at the boundary may generate many different labels. For these sub-windows, the corresponding weight vector can be determined by combining the basic activity segmentations identified before, and the corresponding probability can be obtained. By setting the threshold, the unknown class is rejected, and the classification and recognition are carried out to determine the activity category of the sub-window.

**Figure 9:**
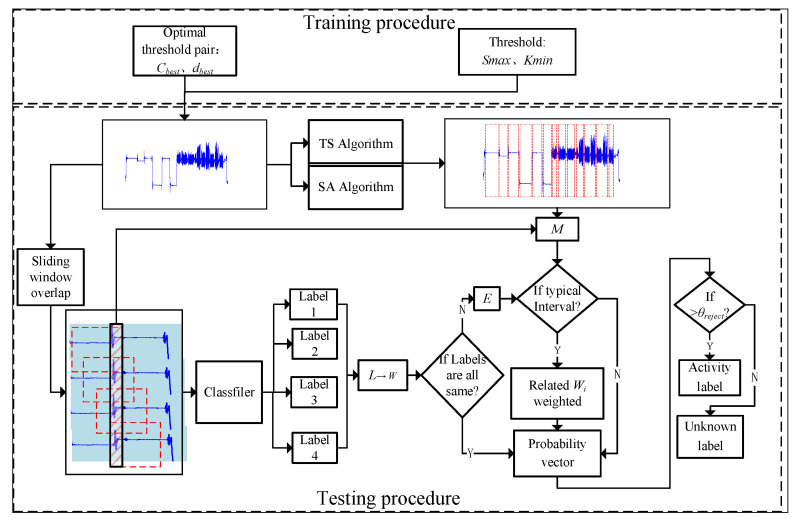
The diagram of the proposed MLWP algorithm.

Let $E=\left\{{s_{m_{1}n}}_{{}_{1}},s_{m_{2}n_{2}},{s_{m_{3}n}}_{{}_{3}},s_{m_{i}n_{i}},\ldots ,s_{m_{k}n_{k}}\right\},1\leq m_{i}<n_{i}\leq t,1\leq i\leq k$ be the set of all abnormal segmentations in the time series, and $l_{m_{i}n_{i}}=\left\{l_{1},l_{2},l_{3},l_{4}\right\},1\leq i\leq k,l\in A$ be the four labels of $s_{m_{i}n_{i}}$, which is generated by the classifier. Let $w_{m_{i}n_{i}}={\left[w_{1},w_{2},w_{3},\ldots ,w_{N}\right]}^{T}$, $w_{m_{i}n_{i}}$ be the weight vector of all kinds of activities from the time interval $m_{i}$ to $n_{i}$, and its initial value is zero vector with size N×1. *N* is the number of activity classes in the time series. Let *M* be the set of all static and periodic-like segmentations according to the proposed TS and SA algorithms. The algorithm diagram is shown in Figure 9. Through the proposed TS and SA algorithms, the thresholds $K_{min}$, $S_{max}$, $c_{best}$ and $d_{best}$ are obtained, and the time series of the test set is pre-segmented. The time series employ overlapping sliding windows to go through the classifier. According to label, *L*, generated by the sub-windows, the corresponding weight vector, *w*, is estimated. When the labels are not completely consistent, the segmentations are identified as *E*, and the segmentation inside the sub-window is judged if it is in *M*. The corresponding *w* is weighted, and the detailed weighting procedure is shown in Algorithm 3. Here, *w* is converted into the corresponding activity probability vector, *P*, using Equation (11). The maximum probability of activity is found, and the threshold, $\theta_{reject}$, is determined to distinguish between the known and the unknown activity.

(11) $$P_{i}=\frac{e^{w_{i}}}{\sum_{i=0}^{z}e^{w_{i}}},1\leq i\leq z$$

For the selection of threshold $\theta_{reject}$, this paper selects a group of complete time series subjects as validation data from the training samples, and the candidate value of $\theta_{reject}$ is set from [0, 1]. Regardless of the accuracy, the maximum is selected as the $\theta_{reject}$ of the data set to reject the unknown activities in the time series.

Algorithm 3 shows the detailed steps of the proposed MLWP algorithm. The output, Lforcast, is the final generated label of *E*.
**Algorithm 3** The proposed MLWP algorithm.**Input:***E, L, M, N, k***Output:***Lforcast*1:**function**weighting(*L*)2:    **for** *i* from 1 to 4 **do**3:        **if** $L_{i}=j$ **then**4:           $w_{j}=w_{j}+1$, 1≤j≤N5:        **end if**6:    **end for**7:    **return** *w*8:**end function**9:**function**weightofcutinterval(x,*w*,*M*)10:    **if** s(x)⊂M && L(x)=j **then**11:        w(x)=w(x)+1.512:    **end if**13:    **return** *w*14:**end function**15:**for***i* from 1 to *k* **do**16:    w(i)=weighting(L(i))17:    w(i)=weighto f cutinterval(i,w,M)18:    Calculate p(j) using Equation (11)19:    *u* = argmax(p(i)),1≤u≤N20:    **if** $u>\theta_{reject}$ **then**21:        Lforcast(i)=u22:    **else**23:        Lforcast(i)=unkonwn24:    **end if**25:**end for**26:**return***Lforcast*

## 4. Performance Evaluation

### 4.1. Experimental Environment and Data Sets

The experiment was conducted on a laptop equipped with AMD Ryzen 5 4600H 3 GHz CPU and NVIDIA GeForce^®^ GTX 1650 2G GPU. The operating system was Windows 10. MATLAB R2019b was used for HAR.

The data sets used in this paper include the UCI and PAMAP2 data set. The UCI (UCI-Rvine, University of California, Irvine) data set comes from “Human activity recognition using smart phones” in the machine learning repository [9]. The data set consists of 30 volunteers aged 19–48 who wore a smartphone (Samsung Galaxy S II) around their waist. Each volunteer performed six consecutive activities (walking, walking upstairs, walking downstairs, sitting, standing, and lying down). Using its embedded accelerometer and gyroscope, it samples 3-axis acceleration and 3-axis angular velocity at a constant rate of 50 Hz. The PAMAP2 data set is measured by nine volunteers wearing inertial measurement units (IMUs) consisting of gyroscopes, magnetometers, an accelerometer, temperature, and heart rate sensor composition. Each volunteer performed 12 consecutive activities [31]. As described in the previous section, this data set was preprocessed, and the sensor data of one experimenter were randomly selected as the verification set, while the sensor data of the other experimenters were used for model training and hyperparameter tuning.

### 4.2. Evaluation Indicators

The problem to be solved in this paper is to accurately detect the starting point of each activity for complex activity time series. In order to evaluate the performance of the proposed scheme from multiple perspectives, the evaluation indicators include accuracy, precision, recall and F1-score [32]. Accuracy represents the percentage of the correct prediction results in the total sample; precision is for the prediction results, which means the probability of actually being a positive sample in all the predicted positive samples; recall is for the original sample which means the probability of being predicted to be positive in the actual positive sample; and the F1-score considers both precision and recall to make both reach the highest at the same time and maintain the balance. The indicators can be obtained as Equations (12)–(15).

(12) $$\mathrm{Accuracy}=\frac{TP+TN}{TP+TN+FP+FN}$$

(13) $$\mathrm{Precision}=\frac{TP}{TP+FP}$$

(14) $$\mathrm{Recall}=\frac{TP}{TP+PN}$$

(15) $$F1-\mathrm{score}=2\times \frac{\mathrm{Precision}\times \mathrm{Recall}}{\mathrm{Precision}+\mathrm{Recall}}$$

### 4.3. Experimental Results

#### 4.3.1. Static and Period-like Interval Segmentation

**Figure 10:**
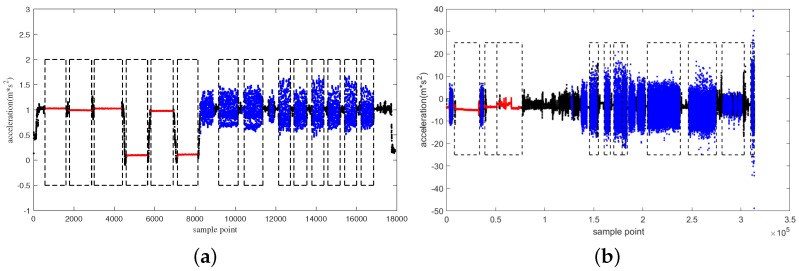
Segmentation results of the proposed scheme in different data sets, (**a**) UCI data set and (**b**) PAMAP2 data set.

In this paper, 5 groups of 60 samples from 30 volunteers in UCI data set were randomly selected as test samples, while others were used as training samples. One of the nine volunteers in the PAMAP2 data set was randomly selected as the test sample, while others are used as training samples. According to the proposed method, the optimal threshold was calculated to identify segmentations. The results of the segmentations are shown in Figure 10.

Figure 10a,b shows the complete continuous activity time series in UCI and PAMAP2 data sets, respectively. The red and blue parts are the static and periodic-like segmentations identified by the proposed algorithm, respectively. The black-dotted rectangular boxes are the manually labeled periodic-like and static segmentations. It is clear that the typical segmentations in the original time series can be clearly figured out.

#### 4.3.2. Model Classification Results

The selection of sliding window size has a certain influence on the final recognition rate [33]. In [34], the sliding windows of 0.5 s, 1.28 s, 2.56 s, and 3 s were selected as the candidate windows on the UCI data set. The final result was that the size of 2.56 s showed the best performance. Therefore, the training set adopted the window of 2.56 s to collect six basic action signals and extract the corresponding time–frequency domain characteristics.

Since the sampling frequency of PAMAP2 data set is 100Hz, this paper selects 1.28 s, 2.56 s, 3.84 s, 5.12 s, and 6.4 s as the size of the sliding window, respectively. The experimental results are shown in Table 3, where 5.12 s performs the best, so 5.12 s is selected as the size of the sliding window on the PAMAP2 data set.

**Figure 11:**
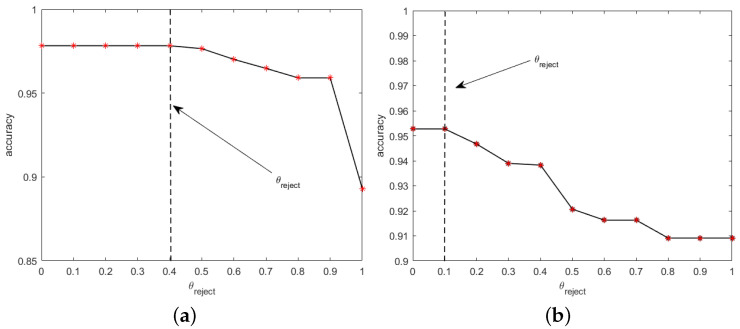
Accuracies of two data sets at different thresholds. (**a**) UCI data set, and (**b**) PAMAP2 data set.

For the UCI and PAMAP2 test sets, the sliding windows of 2.56 s and 5.12 s are used respectively, and 25%(q) overlap is set for feature extraction. The multi-class labels are obtained through the classifier. Among them, the UCI data set involves the identification of the transition actions. Therefore, according to the previously identified static segmentation, the transition action segmentations are derived by the change of before and after actions. For the UCI data set, Anguita et al. proved that SVM had the best performance results, and multi-class labels were obtained by SVM. For the PAMAP2 test set, multi-class labels are obtained by different classifiers trained using training set. The corresponding $\theta_{reject}$ is obtained according to the training set, as shown in Figure 11. Among them, 0.4 is selected as the threshold for the UCI data set, while 0.1 is selected as the threshold for the PAMAP2 data set. The $\theta_{reject}$ of the data set is low since when the PAMAP2 data set is manually labeled, the transition between actions is not considered (the previous sampling point is walking, and the next sampling point is seating), so it is not as good as what the UCI data set shows. The proposed MLWP algorithm is used to determine the label, and the results are compared with those of the manually labeled ones. The experimental results are shown in Table 4 and Table 5.

As shown in Table 4, five groups of test samples are randomly selected, where $S_{1}$−$S_{5}$ represent the first to the fifth groups of data in the test samples, respectively. The highest accuracy is 98.28%, while the lowest is 97.39%. The average accuracy can achieve 97.71%.

As shown in Table 5, SVM, DT, linear discriminant (LDA), NB, KNN and bag tree (BT) classifiers are applied. The accuracies of SVM, LDA, KNN and BT are relatively better than the others, while SVM performs the best, reaching 95.93%.

**Figure 12:**
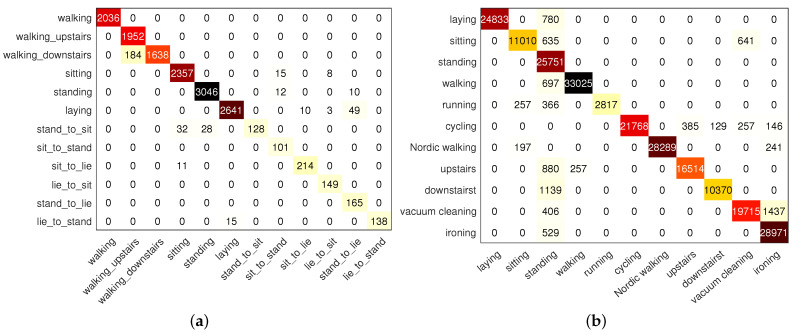
The confusion matrix of the proposed scheme on different data sets. (**a**) UCI data set, and (**b**) PAMAP2 data set.

Figure 12 shows the confusion matrix of the proposed scheme on the UCI and PAMAP2 data sets. From Figure 12a, it can be seen that the classification effect of three types of static activities, sitting, and lying, and three types of dynamic activities, walking, upstairs and downstairs, is very good, while the effect of transition activities (standing to lying, standing to sitting, sitting to standing, sitting to lying, lying to standing, and lying to sitting) is relatively poor because the boundary part is often mistakenly classified into static actions. Additionally, Figure 12b shows that the model has a good recognition rate for lying, running, cycling, walking, going up and down stairs, and relatively poor recognition for sitting, standing, scalding, cleaning and other actions (the volunteer does not perform rope skipping).

**Figure 13:**
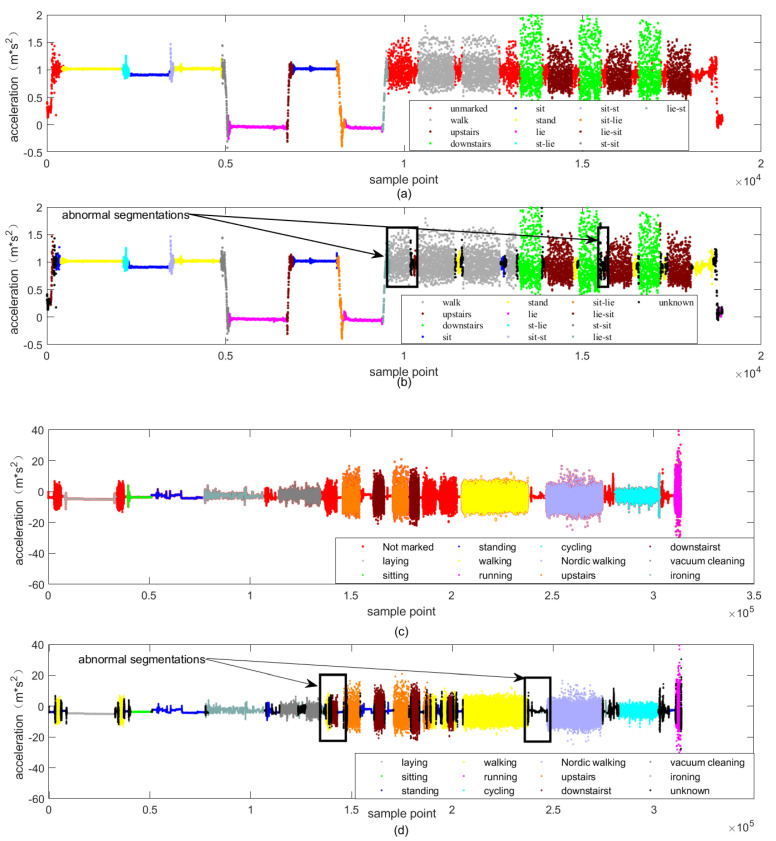
Scatter comparison of x-axis acceleration of the proposed model on different data sets. (**a**) Ground truth of the UCI data set, (**b**) prediction results of the UCI data set, (**c**) ground truth of the PAMAP2 data set, and (**d**) prediction results of the PAMAP2 data set.

As illustrated by Figure 13, different types of actions use different colors; the red spaces in Figure 13a,c is manually unlabeled segmentations, and the black spaces in Figure 13b,d are unknown segmentations rejected according to the proposed algorithm. The first black box in Figure 13b is identified as unknown and walking, because the volunteer may stand up and walk for some time, while the second black box is totally identified as unknown since it can be distinguished as transition action according to before and after actions. Similarly, the first black box in Figure 13d is identified as unknown and downstairs, while the second black box is identified as unknown since it is a transition action. It can be seen that the proposed scheme can clearly segment the time series and identify all kinds of actions. In addition, the unknown segmentation can be distinguished accurately.

**Figure 14:**
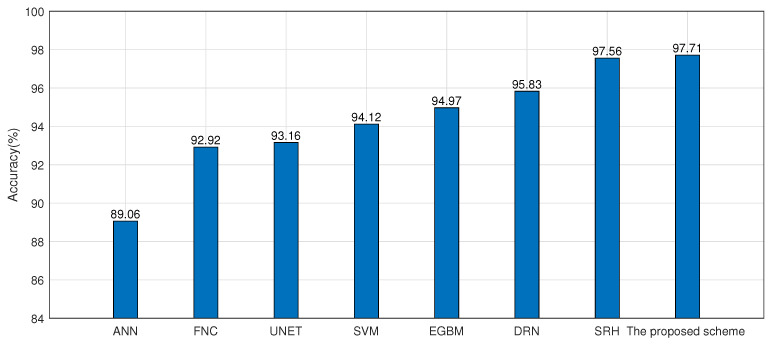
The comparison of accuracy using different methods in the UCI data set.

In order to demonstrate the superiority of the proposed model, this paper compares the results with existing research work. As shown in Figure 14, in [35], the features are first processed by a kernel principal component analysis (KPCA) and LDA. Finally, researchers proposed a deep belief network (DBN) and it was compared with SVM and artificial neural network (ANN). Ref. [36] proposed the U-Net network (UNET) and fully convolutional networks (FCN); UNET achieved fast enough recognition speed. Ref. [37] evaluated extreme gradient boosted machines (EGBM) in HAR. Ref. [38] proposed that shown in Figure 13, a sparse representation based hierarchical (SRH) classifier. Figure 14 shows the comparison of accuracy of different methods in the UCI data set. Numerically, the proposed scheme shows outstanding performance and produces 8.65%, 4.79%, 4.55%, 3.59%, 2.74%, 1.85% and 0.15% higher accuracy compared to that of ANN, FCN, UNET, SVM, EGBM, DBN and SRH, respectively.

Table 6 compares the recall of various types of activities form UCI data sets. Among them, the meaning of A1–A12 is walking, upstairs, downstairs, sitting, standing, lying, standing to sitting, sitting to standing, sitting to lying, lying to sitting, standing to lying, standing to lying, and lying to standing. The proposed scheme produces better recognition results for most of the activities.

**Figure 15:**
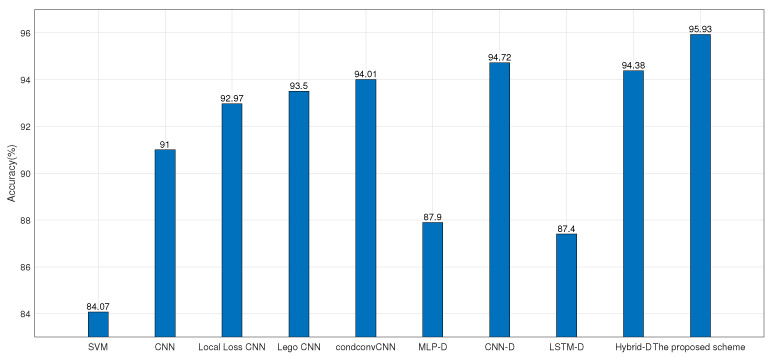
The comparison of accuracy using different methods in the PAMAP2 data set.

For the PAMAP2 data set, the accuracy, precision, recall and F1-core are compared with the existing deep learning-based schemes. As shown in Figure 15, numerically, the proposed scheme shows outstanding performance and produces 11.86%, 4.93%, 2.96%, 2.43%, 1.92%, 8.03%, 1.21%, 8.53% and 1.55% higher accuracy compared to that of SVM, CNN, Local Loss CNN, Lego CNN, condconv CNN, MLP-D, CNN-D, LSTM-D, and Hybrid-D, respectively. As shown in Table 7, the proposed model focuses on shallow learning method. Through probabilistic alignment of the identified typical segmentations, the F1-score is raised to 95.12%. Ref. [39] introduced using the distance-based loss function in MLP, CNN, LSTM and hybrid model, and found that CNN-D shows the best performance among these methods. Compared with CNN-D, the accuracy and F1-score increases by 1.21% and 0.89%. Compared with [26] which introduced condconv to replace the standard convolution layer, the accuracy increases by 1.92%. While compared with [24], which applied the Lego CNN model, the accuracy, recall, and F1-score increases by 2.43 %, 5.64%, and 3.72%, respectively. For the other schemes, the proposed scheme also shows the best performance for the four evaluation indicators. In summary, the proposed shallow learning scheme is able to maintain good classification results with fewer computing resources.

Table 8 compares the recall of various types of activities form PAMAP2 data sets. Among them, the meaning of B1–B11 is lying, sitting, standing, walking, running, cycling, Nordic walking, upstairs, downstairs, vacuum cleaning, and ironing. The proposed scheme produces better recognition results for most of the activities.

## 5. Conclusions

Most of the current research work focuses on simple HAR. Here, classification and recognition are based on manually labeled segmentations in time series, without considering the cost of the manually labeled and personal privacy. In this paper, a probability threshold based algorithm for complex HAR is proposed, which can segment and identify the basic actions in complex activity time series. The proposed scheme accurately segments the activities while effectively rejecting the useless segmentations. In addition, the cost of manual labeling can be reduced to improve the efficiency of HAR. The proposed model is applied to the UCI and PAMAP2 data sets for experiment validation. The results show that for the UCI data set, the proposed model can well segment and identify the static, dynamic, and transition activities. Additionally, the useless segmentation can be effectively identified, and the overall accuracy rate is able to reach 97.8%. For the PAMAP2 data set, the proposed model can distinguish the basic activities well, and the overall accuracy is about 95.9%.

This paper only classifies and identifies six basic activities and six transitional activities. The structure of the proposed model used in the experiment can be further optimized, and more detailed comparative experiments can be carried out. In the future work, in order to verify the robustness and practicability of the proposed model, experiments are planned on various data sets, and the developed modules will be applied to deep learning model. 

## Figures and Tables

**Table 1:** Initial feature set of activity recognition.

Characteristics	Expression	Characteristics	Expression
Mean value	AVG	Standard deviation	Std
Mode	*M*	Maximum	Max
Minimum	Min	Skewness	SK
Kurtosis	*K*	Gravity Frequency	GF
Frequency Variance	FV	Mean Square Frequency	MF

**Table 2:** Description of 14 signals of HAR.

Signal	Description	Signal	Description
$A_{x}$	Acceleration of *x*-axis	$A_{y}$	Acceleration of *y*-axis
$A_{z}$	Acceleration of *z*-axis	$G_{x}$	Angular velocity of *x*-axis
$G_{y}$	Angular velocity of *y*-axis	$G_{z}$	Angular velocity of *z*-axis
$A_{x}^{{}^{\prime }}$	Data difference of $A_{x}$	$A_{y}^{{}^{\prime }}$	Data difference of $A_{y}$
$A_{z}^{{}^{\prime }}$	Data difference of $A_{z}$	$G_{x}^{{}^{\prime }}$	Data difference of $G_{x}$
$G_{y}^{{}^{\prime }}$	Data difference of $G_{y}$	$G_{z}^{{}^{\prime }}$	Data difference of $G_{z}$
$R_{A}$	Resultant acceleration	$R_{G}$	Resultant angular velocity

**Table 3:** The accuracy of the classifier under different window lengths in the PAMAP2 data set.

Window length (s)	1.28	2.56	3.84	5.12	6.4
Accuracy (%)	94.8	94.5	95.2	96	95.9

**Table 4:** Experimental results of five groups of test samples in the UCI data set.

	Accuracy (%)	Precision (%)	Recall (%)	F1 (%)
$S_{1}$	97.48	94.41	94.94	93.93
$S_{2}$	97.73	95.33	90.84	92.72
$S_{3}$	97.39	91.52	96.75	93.47
$S_{4}$	97.68	91.67	91.30	90.51
$S_{5}$	98.28	92.75	96.04	94.08

**Table 5:** Experimental results of the PAMAP2 data set using different classifiers.

	Accuracy (%)	Precision (%)	Recall (%)	F1 (%)
SVM	95.93	93.94	96.71	95.12
DT	81.20	78.23	75.96	74.19
LDA	93.77	91.53	94.21	92.64
NB	85.52	80.85	80.72	88.23
KNN	91.66	89.62	92.25	90.69
BT	95.21	93.30	96.04	94.44

**Table 6:** The comparison of recall of 12 types of activities using different schemes in UCI data set.

Method	ANN [35]	FCN [36]	UNET [36]	SVM [35]	EGBM [37]	DBN [35]	SRH [38]	The Proposed Scheme
A1	83.27	95.77	95.56	88.78	97.78	94.69	98.59	**100**
A2	95.48	93.84	95.54	97.30	96.82	97.12	98.30	**100**
A3	96.88	93.10	91.19	97.61	93.57	97.61	97.86	**100**
A4	91.93	90.43	91.65	95.97	93.89	95.97	97.96	**98.37**
A5	93.99	93.80	94.17	97.58	95.86	97.78	**97.93**	96.88
A6	85.71	95.53	97.20	97.14	98.70	96.67	**99.07**	97.16
A7	34.78	71.43	77.14	73.91	62.86	82.61	82.86	**94.67**
A8	00.00	66.67	75.00	80.00	83.33	80.00	**83.33**	80.6
A9	56.25	83.33	77.08	50.00	91.67	81.25	93.75	**100**
A10	76.00	84.85	75.76	64.00	81.82	72.00	87.88	**100**
A11	51.02	85.71	83.67	69.39	75.51	85.71	87.75	**100**
A12	18.52	81.58	71.05	62.96	73.68	81.48	84.21	**84.43**

**Table 7:** The comparison of evaluation indicators using different methods in the PAMAP2 data set.

Method	SVM [11]	CNN [11]	Local Loss CNN [40]	Lego CNN [24]	Condconv CNN [26]	MLP-D [39]	CNN-D [39]	LSTM-D [39]	Hybrid-D [39]	The Proposed Scheme
Accuracy	84.07	91	92.97	93.5	94.01	87.9	94.72	87.4	94.38	**95.93**
Recall	84.71	91.66	-	88.17	-	-	-	-	-	**93.94**
Precision	84.23	91.54	-	91.07	-	-	-	-	-	**96.71**
F1-Score	83.76	91.16	93.03	91.4	-	86.66	94.23	86.53	93.88	**95.12**

**Table 8:** The comparison of recall of 11 types of activities using different schemes in PAMAP2 data set.

Method	CNN [40]	Local Loss CNN [40]	Lego CNN [24]	DanHAR [25]	The Proposed Scheme
B1	90.3	90.3	90.3	90.3	**97**
B2	98.4	97.8	**98.4**	95.1	90
B3	86.3	92.3	92.6	93.7	**100**
B4	35.9	50.3	58.0	47.3	**98.0**
B5	96.5	97.8	**98.2**	96.9	81.9
B6	94.6	94.1	73.5	94.1	**96**
B7	86.4	93.8	94.3	95.5	**98.5**
B8	98.1	98.1	**99.0**	97.1	93.6
B9	91.6	94.4	88.8	**96.2**	90.1
B10	83.2	87.4	84.2	79.8	**91.5**
B11	88.3	91.6	94.7	95.5	**98.2**

## Data Availability

Not applicable.
